# An archive that will have moved: on an exemplary artistic intervention in the archive Fritzpunkt, its relation to the Marianne Fritz archive in Vienna and its conceptual ramifications

**DOI:** 10.1007/s10502-026-09565-6

**Published:** 2026-10-07

**Authors:** Lukas Schmutzer

**Affiliations:** University of Vienna, Vienna, Austria

**Keywords:** Archive art, Archive Fever, Marianne Fritz, World War I, Poetics, Historiography, Mikhail Bakhtin

## Abstract

In 2023, the *Agency for Indispensability Justifications* (AGFU) sent shredded objects to 271 archives worldwide. This was part of a larger project of developing an *Aufhebekunst* [art of sublation] by engaging with the remains of the former artist collective *Fritzpunkt*, namely: the *Archive Fritzpunkt.* For years, *Fritzpunkt* had performed *The Fortress*, a neo-avant-garde work by Austrian author Marianne Fritz about Galicia during World War I, in what is today Ukraine and Poland. Inspired by Fritz’s writing, *Aufhebekunst* subsequently performed the *Archive Fritzpunkt*, ultimately causing it to disappear. This article does not only situate the AGFU’s provocative ventures within the tradition of archive arts. Building on Mikhail Bakhtin’s methodology, this article understands the AGFU’s artistic performance as a transfer from the particular linguistic performance developed in The Fortress to the Archive Fritzpunkt. Therefore, the AGFU’s engagement with concepts associated with the archival turn are compared with strategies by which these concepts are applied to poetic texts. Particular attention is devoted to Jacques Derrida’s Archive Fever, whose underlying linguistic concept is contrasted with the demands that The Fortress places on its own reception. Finally, a case study investigates the poetic structure of The Fortress in regard to Fritz’s appropriation of archival records from the Austrian State Archives. Thus, the article argues that Fritz’s poetic practice includes a particular mode of reading archival records. The AGFU’s Aufhebekunst applies it to archiving itself and thus addresses as well as provokes the archival ethos.

There is no work of art that does not appeal to a people who do not yet exist.

(Gilles Deleuze^1^)

## Introduction: *The Archive is Present* and the AGFU

Standing in the middle of the installation *The Archive is Present. Oder Lob der Lücke* [The Archive is Present. Or Praise for the Gap^2^], a professional archivist reported an urge to organise the objects. But then again, he said, he could see another kind of order: a landscape. And in fact, the objects were arranged in such a way that one could wander through them, allowing certain paths and prohibiting others. They were stripped of any context, except for a label indicating their dimensions, weight and affiliation with the project from which they emerged. In the former library of the old Vienna University of Economics and Business, the *Agentur für Unabkömmlichkeitsbegründungen* [Agency for Indispensability Justifications]) (AGFU) had installed the archive of the former Austrian artist collective *Fritzpunkt.* In the 15 years from 2002 to 2017, *Fritzpunkt* created installations and forms of performative and theatrical appropriation. Those activities led to the inadvertent creation of the *Archive Fritzpunkt* (AF), spanning 25 domains and covering numerous object categories ranging from audio, video and photo storage media, to handwritten working notes, concept papers, literature, props from performances, to e-mails and bills. After the end of *Fritzpunkt*, the transfer of the remaining items to a more established institution was considered. But, instead, in 2019 *Fritzpunkt* was converted to the AGFU.

Having full access to the AF, the AGFU’s primary objective was to develop an *Aufhebekunst* [art of sublation] (Fritz 1996, p. 80) that focuses on paradoxical archiving techniques. The AF should reveal its wealth by transforming its objects into performative installations and ultimately disappear, while the *Aufhebekunst* should provide techniques that can be transferred to other archives. The term exploits the ambiguity of the German word ‘aufheben’ [to sublate] in the sense of ‘to store’, ‘to abolish’ and ‘to raise’. In our context, it was used following Austrian author Marianne Fritz (1948–2007), whose work was the main concern for *Fritzpunkt* and thus for the archive being dealt with. The three main works of her large project *The Fortress*, *Dessen Sprache du nicht verstehst* [Whose Language You Do Not Understand] (Fritz 1985), *Naturgemäß I* [Naturally I] (Fritz 1996) and *Naturgemäß II* [Naturally II] (Fritz 1998), were published by Suhrkamp. Together with two other published works and the fragments of the estate, this unfinished cycle comprises almost 10,000 pages. In *Fritzpunkt’s* self-description, it inspired the collective to investigate the canon of theatre forms for more than a decade. Characterised by an experimental approach to archives, *The Fortress* examines Galicia in the First World War, “a centuries-long deployment zone of European and Asian armies” (Kastberger/Neundlinger 2012, p. 9) in today’s Ukraine and Poland. In one of her poetological texts, Fritz argued: “We must learn to understand our paradoxes, not repress them, not reassure them, we must learn to sublate them within us[.]” (Fritz 1994, p. 25) This argument has to be understood in relation to Critical Theory and especially in the context of Walter Benjamin’s reflections on allegorical textual exegesis, which “raises” the things that signify – signs “pointing to something else” – “onto a higher plane” (Benjamin 2003, p. 175) and which was understood as the basis of a theory of montage among the avant-gardes (Bürger 1984, pp. 69–82).

The focal point of *Aufhebekunst* was the installation *The Archive is Present* in the summer of 2023. Conceived in three parts, the remaining objects of the AF were first installed and made open to the public for three days as an *Archive of Presence*. They were marked out by an array of white lines drawn on the floor. For the second part, the *Archive as Fragment*, all objects were shredded and the shreds were returned to their former places. For five days, each object was exhibited again, though it had been transformed: some of the objects now exceeded their previous boundaries, such as the piles of paper, while others did not even come close to their previous dimensions.

Afterwards, each object was sent to one of 271 archives around the world, together with a short description of the project and the request to include “the migrant without permit” into the respective archive. The AGFU started its selection process with an examination of the directory of the Verband deutscher Archivarinnen und Archivare [Association of German Archivists] (2021). For international addresses, the AGFU undertook “time-consuming individual enquiries” into lists such as that of the members of the International Centre for Archival Research.

The responses turned out to be diverse, ranging from a request to recuperate the special waste to simple acknowledgments of the *Swiss Archives of Contemporary History* (Nerlich/Schärli 2024, p. 18), as well as posts on social media. The *State Archives of Hessen* has coined the term “Archivalienkunst” [archival documents art] for the object sent to them (Moriandi 2023).

In the meantime, the entire installation was cleared except for the array of white lines on the floor: what had initially measured the objects was now the only trace left of them. Thus, an *Archive of Absence* was created that remained open to the public for nine days. Finally, in this very space, the symposium *Whether or how to document a self-dismantling archive?* brought together archivists, philosophers, literary scholars and art historians.

**Figure:**
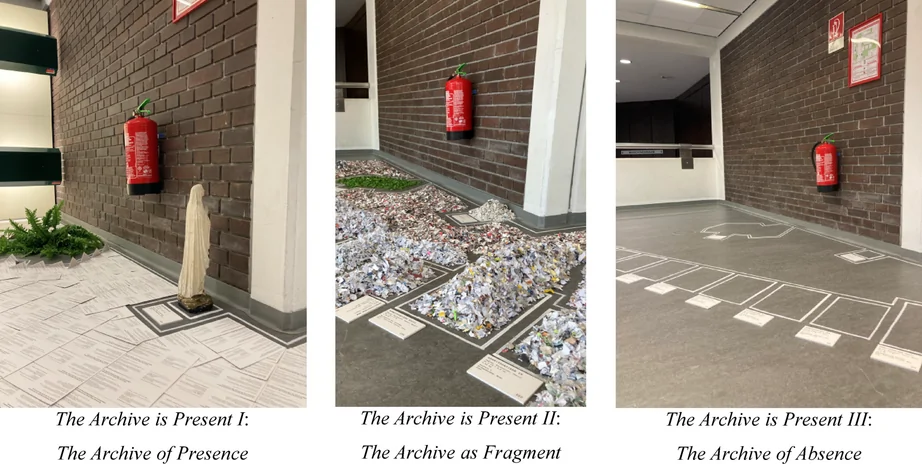
Taking photos was “not recommended” by the AGFU. It should be noted that there was a tough debate with the AGFU about whether the photos presented in this article could be justified in the context of *Aufhebekunst*.

## Approach and outline

Alluding to the performance by Marina Abramovic from 2010, *The Artist is Present*, *The Archive is Present* transformed the AF into “a dynamic and self-reflective medium that intervenes in and challenges its own ontology” (Borggreen and Gade 2013, pp. 25 − 6). This is what the notion of “performing archives” (Borggreen/Gade 2013, p. 25) or indeed the “critical archive” (Danbolt/Spieker 2014) suggests. The archival ontology is addressed in different ways throughout the various stages of *The Archive is Present*: the array of white lines opening up a landscape to the visitors also hints at the transcendental problem of an ordering grid (Krauss 1979/Spieker 2008, p. 9). The fragments do remind us that classical archival thinking is tied to “the experience encapsulated within” the objects (Lustig 2020, p. 76). The absence gestures in a radical way towards the transcendental relation of the ordering grid to the objects to be ordered (Steedman 2001, pp. 152-4). The shipping of the archival objects irritates the concept of administrative bodies designed for “staying and storing” (Plener 2020).

By signifying an archival document as a “migrant without permit”, a word from a specific context is ‘raised’ – or ‘sublated’ – onto another plane. On the one hand, this performative intervention can be compared to artistic strategies of intertwining spaces that were initially foreign to each other, as done in installations of Guillaume Bijl or Christoph Büchel. On the other, it can be described in linguistic terms as an intervention in the poetic function of communication. Instead of deducing an equation by sequencing words, in poetic speech an “equation is used to build a sequence” (Jakobson 1981, p. 27) and “addresser and addressee become ambiguous” (Jakobson 1981, p. 42), together with the words themselves. Thus, by the AGFU’s equation ‘record = migrant’, the object’s status and its provenance is questioned as well as the receiver’s relation to it.

In each of its stages, *The Archive is Present* shifts the focus from epistemological aspects to an investigation of what might be termed the “relational potential” of objects (Massumi 2011, p.52). The landscape of *The Archive is Present* as well as the violation of the integrity of the objects provoked questions about the individual experience in the very moment of the visit. The shipping of the archival shreds created a network of relations. On the ground floor of the former library of the old Vienna University of Economics, which was opened especially for the *Archive of Absence*, the real-time status of the shipments was projected onto an installation of ashlars. Distorted by their three-dimensional structure, visitors could read about the objects, the addressees and whether the parcels were “en route”, “delivered” or “returned”.

This relational logic explicates the distinctive nature of *Aufhebekunst* and sets it apart in the field of archive art. Observing the *archival turn’s* reception in the arts, Sara Callahan speaks of a “structural similarity” between a “theorized notion of the archive” and “the institutional art world” (Callahan 2024, p. 76; see also Callahan 2022, pp. 88–94). According to this similarity, works of art “essentially are that institution” (Callahan 2022, p. 191): the AGFU’s intervention within the AF fundamentally criticises this analogy. Here, *Aufhebekunst* provokes in order to escape the archives by performing an institutional framework, which relates the AF as a “secondary archive” – in the AGFU’s terms – to a “primary archive”: The *Marianne Fritz Archive* in Vienna, which was built from the working methods created for *The Fortress* and which contains the author’s legacy today (Kastberger/Neundlinger 2012).

This article derives the artistic performance of *Aufhebekunst* from linguistic performance. My methodological approach assumes that the AGFU’s attitude towards archival material can be perceived by reflecting upon what Mikhail Bakhtin called the “addressivity” (Bakhtin 1986, p. 95) of words. This term assumes the speakers’ relations as a basic principle of the formal structure of language. It derives from Bakhtin’s concept of the “speech genre”, which can be compared to the “speech act” respectively the “performative” in John L. Austin’s theory (Austin 1962).

I argue that the AGFU’s approach stems from the *Fritzpunkt* collective’s readings of *The Fortress*, since the idiosyncratic poetic structure of Fritz’s texts provokes reflections on the relation of its various speakers. Their voices arise, at least in major parts, from an engagement with the linguistic forms of archival records from the Austrian State Archives. In other words, following Bakhtin’s concept, I assume a specific form of reading that we may call ‘performative’ and that was conducted by Fritz and transferred by the AGFU into artistic performance. The AGFU also applied this kind of reading, as I argue, to texts associated with the *archival turn*, which inspired *Aufhebekunst*.

After an overview of the AGFU’s installations in the context of archive art, I will reconstruct this performative way of reading by looking at how poetic texts are read and understood in the course of the *archival turn*. Apart from two examples concerning literary texts, I will provide a critique of Jacques Derrida’s *Archive Fever*. As an essential text in the *archival turn* it is apt, not only because it develops a concept of the archive through a specific linguistic performance that reflects poetic texts, but also because it accounts for destruction and forgetting as irreducible constituents. This aspect establishes the connection between *Aufhebekunst* and the working methods of Marianne Fritz, which are analysed in the subsequent section. A case study examines one form of Fritz’s appropriation of archival records in *The Fortress*, before the conclusion turns this mode of reading back onto *Aufhebekunst* and builds a bridge to archival science.

## From the *disaster of the archive* to the *Aufhebekunst*

In September 2019, the title of the AGFU’s inaugural installation at Galerie Krinzinger Schottenfeld in Vienna brought together two concepts: *Desaster des Archivs oder Arbeit am Verschwinden* [Disaster of the Archive or Work on Disappearance]. Ebeling uses the notion of the “disaster of the archive” to confront the archive as a transcendental figure with the problems of bearing witness that Georges Didi-Huberman discusses when dealing with documents from Auschwitz: “After all, there are not only the archives of disaster that Didi-Huberman evokes; there is also the disaster of the archive[.]” (Ebeling 2007, p. 34f) While Ebeling’s ‘turn’ is excluded from the AGFU’s title, it is present implicitly in the relation of the AF to *The Fortress* and the ‘primary archive’ from which it emerged. At its core, *The Fortress* addresses the Holocaust in the most complex forms (Kastberger/Neundlinger 2012, p. 12), and hence it reflects on issues of representation where evidence remains only “a fracture, a discontinuity, the mark of which is obliteration, erasure and amnesia” (Merewether 2006, p. 12). Looking at the archives of twentieth century’s catastrophes, Okwui Enwezor criticises Adorno’s dictum on the barbarism of writing poems after Auschwitz, concluding that art “cannot be excluded as a way to give language and word to the unspeakable” (Enwezor 2000, p. 20). However, this dictum could also be read not as a criticism of poetic language but rather of its classical forms. In order to produce progressive forms, “art can do no better than close its eyes and grit its teeth” (Adorno 1997, p. 321).

By adopting a negative stance in Adorno’s sense, the AGFU engages in a radical critique of the “Archival Impulse” (Foster 2004) in contemporary art. According to Callahan, Foster also introduced this notion to emphasise the critical potential of the neo-avant-gardes in response to Bürger’s criticism that they had been “co-opted and taken up as mainstream” (Callahan 2022, p. 191; see also pp. 23 − 4 and Foster 2001). Austria provides an exceptional situation, where the avant-garde and neo-avant-garde coincide: “Owing to its fundamental lack of national esteem and the future prospects following the First World War and the end of the multi-ethnic Habsburg monarchy, there was a tremendous delay in the historical avant-garde reaching Austria[.]” (Tezarek/Zolles 2021, p. 332). Predicated on Gerhard Rühm’s gesture of forestalling the Wiener Gruppe’s own institutionalisation, Kastberger argues that the Wiener Gruppe was “conceived from the very beginning as an archive of itself” (Kastberger 2023, p. 55). As a striking example, he refers to the piano key that Rühm preserved for later generations after he had ripped it out during a performance in which a piano was destroyed (Kastberger 2023, p. 56). Thus, Kastberger argues, the great “poetic act of the Austrian avant-garde” was to create an archive and thus to have poetically transformed ‘the’ archive itself (Kastberger 2023, p. 66). However, this specific Austrian archival impulse is ironically subverted by the institutional play between the AGFU and the AF: *Aufhebekunst* radically questions whether – and if so, under what conditions – documentation can ever be poetic.

The radicality of this negative approach is exemplified by the AGFU’s earlier projects. As part of the first installation and its successor in Vienna’s Bräunerstraße, *Strategien zur Archivvermeidung* [Strategies for Avoidance of Archives], the AGFU produced multiples: 5,800 pages of working notes from the AF were transformed into 26 epoxy resin objects of 220 pages each. In these multiples, documents are simultaneously preserved and destroyed. They could be considered as the exteriorisation of partial forgetting (Assmann 2016, p. 18): They constantly remind us that something has been there and at the same time withdraw it from our grasp. An erasure cinema that was installed in the same location at Bräunerstraße is another example of the various, paradoxical methods developed by the AGFU. Fritzpunkt’s e-mails were erased from their hard-drives with the most thorough method possible: The *Gutmann method* swipes 35 layers of 0s and 1s over the section to be deleted (Gutmann 1996). This process accompanied the entire installation, which took place from 8th to 15th November 2021. It was projected onto the walls, while a lounger provided a place to lie down and to watch the mail titles disappear together with the processing of the layers. This performative installation reminded visitors that data, as the “perceived opposite” to materiality (Callahan 2022, p. 114), is a product of media processes and that its erasure is merely a form of transformation from one code into another.

**Figure:**
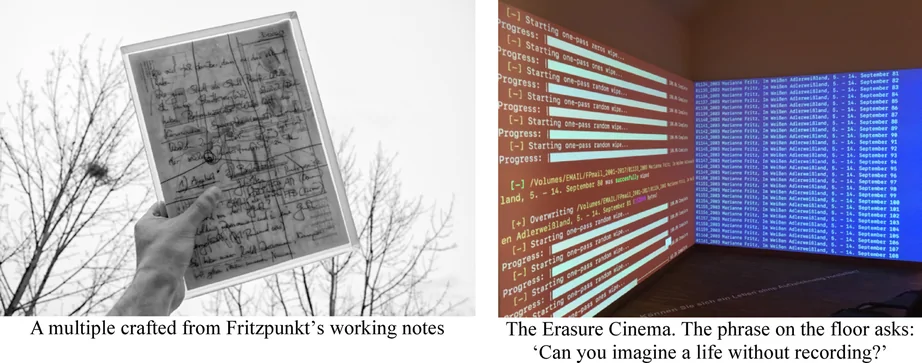

Those interventions can be seen in a tradition of resistance against the “model of the archive” that was developed by bureaucratic rationalisation (Sekula 1986, p. 58). However, they must be distinguished from an approach that foregrounds exclusions from hegemonic archives “by presenting them ‘as yet’ another archive” (Van Alphen 2015, p. 123). Subsumed under the “invention of counter-archives” (Enwezor 2008, p. 22), the AGFU’s interventions are still designed as a means to escape the archives. The AGFU assumes that there might be a way of approaching documents that is non-archival and perceives absence as a potential.

Furthermore, *Aufhebekunst* expounds problems of ephemeral art in regard to its own documentation: “According to the logic of the archive, performance is that which does not remain” (Schneider 2001, p. 100), because it “becomes itself through disappearance” (Phelan 1993, p. 146). Schneider highlights the performer’s corporeal experience as another kind of archive. This notion does not apply to the object-centred nature of *Aufhebekunst*. Taylor’s distinction between the archive and the repertoire, with the former constituted of “materials that seem to endure” (Taylor 2007, p. 19) and the latter as embodied memory with its “own structure and codes” (Taylor 2007, p. 20), is questioned by the institutional framework between the AF and the Marianne Fritz Archive, which each combine aspects of both notions. As Tancredi Gusman argues, documentation can aim at the traces of an event that has been, in order to keep it in evidence, or it can aim at a representation of aesthetic qualities to preserve their effect (Gusman 2019). However, by performing the archive, the objects of *Aufhebekunst* question their own evidence and in turn re-present the conditions of the documentation’s own possibility.

It is against this backdrop that my own role within the project is to be situated: as part of a collaboration, I was responsible for documenting each of the AGFU’s ventures, starting with the installation at Bräunerstraße. As a writer and literary scholar I chose to react upon each of the aesthetic encounters with words, thus creating texts that mingle my subjective experience with scientific discourse. The result was a series of essays that are closely interlinked through certain motifs and terms, comparable to “plateaus” (Deleuze/Guattari 1987, pp. 1–2). Together with texts from other project members, the AGFU compiled these essays into a map made of linen (Innerhofer/Brucher/Schmutzer 2024). Fully unfolded, it spans several rooms and requires collaboration between various recipients to lay it out. Thus, the conditions and limitations of documentation inevitably lead to reflection.

## Archives and poetics

Ebeling uses the term “disaster of the archive” in the context of a “philosophy of the archive” (Ebeling 2007, p. 35), which queries the traditional approach of researchers searching for documents and producing knowledge by shaping the records. Famously, in his *Archaeology of Knowledge*, Foucault decentres the place of transcendental inquiry and shifts it from the researching subject to its outside, which he defines by ‘the’ archive as a new methodological concept (Foucault 1972, pp. 126 − 31). Turned back to archival practice, the concept persists that, first and foremost, archives always shape what we can recognise. Ebeling elaborates an aporetic discourse regarding an inaccessible archive as the condition of the possibility for knowledge. Paradigmatically, he describes Kafka’s *Before the Law* as being “its own archive”. The short narration about a man who is refused entry into the law “has recorded its own inaccessibility” (Ebeling 2007, p. 51). This interpretation is reminiscent of the term ‘kafkaesque’, which is often used to describe mysterious, compulsive settings. Burkhardt Wolf recently demonstrated that the “inevitability” typical in Kafka’s texts stems from the author’s expertise in administrative processes (Wolf 2023, p. 317). Kafka was employed at an insurance company in Prague, where his tasks involved processing individual accident claims and transferring them into bureaucratic workflows. In this regard, it can be assumed that Kafka had a keen sense for linguistic performance and how different genres can be played against each other. Anyone who examines *Before the Law* for simple speech acts, and respectively speech genres, finds an implicit objection in the prompt of the narrative’s gatekeeper: “If it tempts you so, just try to enter, despite my prohibition.” (Kafka 1994, p. 267) He forbids entry, at the same time as he encourages it. The aporia in Kafka’s text is not to be understood as an effect of a greater law. It can be accessed by looking at the agonistic set-up of the modes of address.

In a reversed aporia, Sven Spieker describes the protagonists’ quest for universal knowledge in Flaubert’s novel *Bouvard and Pécuchet* as a futile attempt to access an external place that does not exist: “As a storehouse of knowledge, the modern archive refers us to a place outside of itself, the very place Bouvard and Pécuchet are seeking.” (Spieker 2008, p. 33) However, the desire for knowledge of the two protagonists cannot be abstracted from either the narrative mode of the novel or the sources from which they seek to acquire their knowledge. Flaubert’s narration quotes genres of contemporary knowledge transfer and, as Karlheinz Stierle shows, wrests from them an aesthetic reflection that exposes their ideological conditions (Stierle 2012, p. 186). Spieker examines a passage from the beginning of the novel’s fifth chapter, which thematises the knowledge of literature itself. The protagonists discuss modes of representation in relation to information that has no function for the plot and thus represents an excess. Such an excess leads away from the narrative and refers to the systematic nature of scientific discourse – the interplay of these layers in Flaubert’s text encourages reflections on various forms of knowledge. Unlike a “philosophy of the archive”, Spieker does not carve out an archival base layer. Rather, he explores the possibility that “the archive and what it stores emerge at the same time so that one cannot easily be subtracted from the other” (Spieker 2008, p. xi). Here, his reading is considered together with Ebeling’s reading of Kafka to demonstrate how the critique of archival ontologies – in this context of literary analysis – revolves around aporias. These aporias are rather effects than conditions. A shift towards the addressivity of utterances proves the aporias to be communicative or ideological barriers caused by the configuration of speech genres.

The aesthetic analysis focuses not on the hierarchy of layers, but on the forms of their relation. The AGFU transgresses this distinction from the other side. I argue that the artistic performance of a “disaster of the archive” involves a creative misunderstanding which changes the epistemological problem into an aesthetic inquiry. The term “migrant without a permit” has already been noted as a specific use of language. In a similar way, the “disaster of the archive” is not used to deduce equations, but as a starting point for building new forms. While the *archival turn* has claimed that archives shape our knowledge, the AGFU points archives back to their own forms.

Besides Foucault, Ebeling derives the “disaster of the archive” from Derrida’s concept of *Archive Fever* and the archive’s “power of *consignation*” (Derrida 1995, p.10 / Ebeling 2007, p. 86), which stems from founding principles of the European Archive. According to Derrida, it is from this power that the “question of the archive” (Derrida 1995, p. 12) arises, that he addresses in the singular, but frames in different phrases. It concerns the problem of demarcation (“where does the outside commence?”, Derrida 1995, p. 12) or precedence (“what comes first?”, Derrida 1995, p. 28). The complex argument of *Archive Fever* could be portrayed as leading from this question of logical or local hierarchy to “a question of the future” as well as to “the question of a response” (Derrida 1995, p. 27). Here we find Derrida’s sensitivity to pragmatic relations, which he is continuously introducing into theoretical discourse. However, he conceives this in a tragic and patriarchal configuration: *Hamlet* and *Oedipus*, who both are paradigmatic for psychoanalytic concepts (Zizek 2004), serve as a paradigm for a “constitutive relation between memory (loss) and archiving” (Cvetkovich 2025, p. 200). Derrida conceives archives as a “repository or domicile” (Brothman 2025, p. 32) that is threatened by the “violence of forgetting” (Derrida 1995, p. 51) – while *Aufhebekunst* has to be situated in a tradition of critical thinking that perceives forgetting as a productive momentum (Weinrich 2000).^3^ Paul Celan’s verse “Led home into oblivion” (Hamburger/Celan 1971) – to cite a brief example – reverses the psychoanalytical model that seeks to prevent forgetfulness (Lönker 2003, p. 69). According to “Celan’s implicit theory of experience”, a perception “must first sink into the ‘night’ of ‘forgetting’ in order to become a remembrance.” (Menninghaus 1980, p. 93) This argument reveals a double bind in Derrida’s “archiviology” (Derrida 1995, p. 26) to methods of deconstruction as well as to psychoanalytic concepts, both of which are searching for an origin, albeit an impossible one. This is decisive in Derrida’s transcendental inquiry and has continuously influenced his examinations of Freud and “the operation of his writing” (Derrida 1987, p. 284), where “archives […] have been Derrida’s quarry after all” (Brothman 2025, p. 27).

In his early essay *Freud and the Scene of Writing*, Derrida examines Freud’s technical metaphors to describe “the very essence of the psyche” (Derrida 2005, p. 252) as a progression of signs with no permanent code. Grammar and meaning are produced by the force of breachings. Ultimately, Derrida replaces Freud’s metaphors with the archive: “The unconscious text is already a weave of pure traces, differences in which meaning and force are united—a text nowhere present, consisting of archives which are *always already* transcriptions.” (Derrida 2005, pp. 265-6) The notions of “meaning” and “force” are central to Derrida’s take on the performative (Derrida 1988, pp. 1–2 and 9), and the “trace” also marks a concept that stays present in his thinking (Bergo 2014), while archives are of almost no importance in Freud’s work; he uses the term explicitly only once in his writings (Spieker 2008, p. 39 and 201). From this perspective, Derrida’s archive “is the question of philosophy itself” (Naas 2015, p. 140), because it asks for a substrate of a concept of writing that Christoph Menke portrays as transgressing all substrates (Menke 2012, p. 194). It is this question that causes *archive fever*.

## The poetic function of the primary archive

Born in 1948 in Weiz in Styria, Marianne Fritz published her first novel *The Weight of Things* in 1978 (Fritz 2015). The book, which is about a hundred pages long, tells the story of a woman from the lower middle class after World War II. Gradually, Fritz radicalised her avant-garde methods and developed them in ever more expansive books, while going backwards in the history of the 20th century. Her second novel, *Das Kind der Gewalt und die Sterne der Romani* [The Child of Violence and the Stars of the Romany] (Fritz 1980), comprises around 550 pages and depicts a village community after the collapse of the Habsburg monarchy (Fritz 1980). Moved to Suhrkamp, her third novel *Whose Language You Do Not Understand* runs to 3,300 pages and examines the origins of World War I from a proletarian perspective (Fritz 1985). The novel narrates the aggressions that led to the war.

In the context of the entire cycle, the name ‘Fortress’ has so far referred to the psychiatric institution of the Monarchy’s capital. As a literary motif, it implies a relationship between society and its psychic idiosyncrasies. The successor *Naturgemäß* – a word that is difficult to translate, but which is roughly covered by “naturally” – shifts its interest to a military fortress of World War I. It freezes and dissects the siege of Przemysl layer by layer across more than 5,000 pages (Fritz 1996/1998). The work has remained unfinished: Fritz died in 2007 in Vienna (Schmutzer 2023, paragraphs 1–2). The Fragments from the third part of *Naturgemäß* show how the backward movement through history was conceived as a means of progressing along the linguistic forms of the century in order to approach the Holocaust. For example, the text contains one of the most “anti-Semitic inflammatory pamphlets”, Ernst Hiemer’s children’s book *Der Giftpilz* [The Poisonous Mushroom] (Kastberger/Neundlinger 2012, p. 13), which is juxtaposed with Hölderlin’s poem *In my days of boyhood* (Hölderlin 1952, p. 119). The montage techniques developed by Fritz explore the soil of 20th-century hatred. Those techniques can already be sensed in Fritz’s first novel, in which the Monarchy’s capital is not named Vienna, but “Donaublau” [Danube blue] (Schmutzer 2023, paragraph 1). This is not only an ironic allusion to *The Blue Danube* by Johann Strauss II and, with it, to the Habsburg myth (Magris 1988) – but the capital is also being transcribed into a fictional world. In the subsequent books, Donaublau gets entangled in an increasingly dense network of fictional components. Due to these “alienation effects” of montage (Innerhofer 2023, paragraph 3), readers must trace the relations within the text before they can link them to historical contexts. Methods such as these engage with the addressivity inherent in words, enabling *Naturgemäß* not to report on war, but to send the words themselves to war. The Marianne Fritz Archive, located in the author’s former apartment in Vienna, emerged from the author’s working procedures (Kastberger/Neundlinger 2012, p. 18–37). A literary archive to which the materials were consigned would face the problem of how to transfer these processes – which are inherent in the apartment’s architecture – into new boxes. I would like to discuss a brief example to demonstrate these methods in regard to Fritz’s appropriation of archival records.

Page 187 of *Naturgemäß* marks the beginning of a new chapter and offers a new form of narration. Readers are confronted with a montage of two texts: one text is a status report which is sent from the “Nabel der Welt” [Navel of the World] to the commando unit in “Kranichweib” [Crane-woman] (Fritz 1996, p. 187). It is indented and lists what it calls “made experiences” during the siege of a fortress, describing defensive positions in detail and how enemy fire affected them. This list is interrupted by another text, aligned to the left of the page and in another style of lettering, consisting of a stream of words gradually revealed to be voiced by an officer called “der Willensbrecher” (the Will-breaker) (Fritz 1996, p. 189). This is a character from the preceding novel *Whose Language You Do Not Understand* (Fritz 1985, pp. 294–307), while the text is written in a similar, idiosyncratic style that transgresses narratological categories (Schmutzer 2023, paragraph no. 8). The narrator usually stays close to its characters’ consciousness, yet remains highly flexible, shifting perspectives and distances not only between sentences but even between individual words. The characteristics of free indirect discourse – a mode that makes it unclear whether “the words are the character’s words” while it is suggested “that they *might* be” (Chatman 1975, p. 254) – are integral to the style of the text, which is open to the “twilight realm of consciousness” (Cohn 1966, p. 110) and to “lyric and ironic possibilities” (Cohn 1966, p. 104). In this setup, different ways of conveying experiences of war implicitly comment on each other. In the beginning, the status report mentions that the bombardment of the fortress has brought no greater damage, while the speech of the Willensbrecher starts with an account of a projectile striking a place next to him. In contrast to the report, the Willensbrecher refers to friendly fire and reveals how it killed one of his inferiors whom he calls by the nickname “the Thinker” (Fritz 1996, p. 189). The Willensbrecher’s reflections are characterised, on the one hand, by strong and aggressive judgements and, on the other hand, by expressions of erotic desire towards his inferiors. Those two aspects mingle together, for example, when the Willensbrecher distinguishes “human material” (Fritz 1996, p. 191). He attributes first-class status to the murdered “Thinker” and laments that the second-class “Runner” could escape the impact (Fritz 1996, p. 189). At the same time, he desires both of them and ponders how they are “perfected” through him (Fritz 1996, p. 189). The argumentation of the Willensbrecher starts by way of montage in between two words of the status report: “untunlich erscheinen” (Fritz 1996, pp. 187-8), which translates to something appearing ‘inadvisible’ or ‘inappropriate’. With this wording, the report refers to the current reinforcement of the besieged fortress which has to be adapted due to the developments of warfare. Thus, Fritz opens up an inappropriate fortress and inserts a discourse. In an ironic way, the montage asks if this fortified speech is appropriate; if it judges what it desires.

**Figure:**
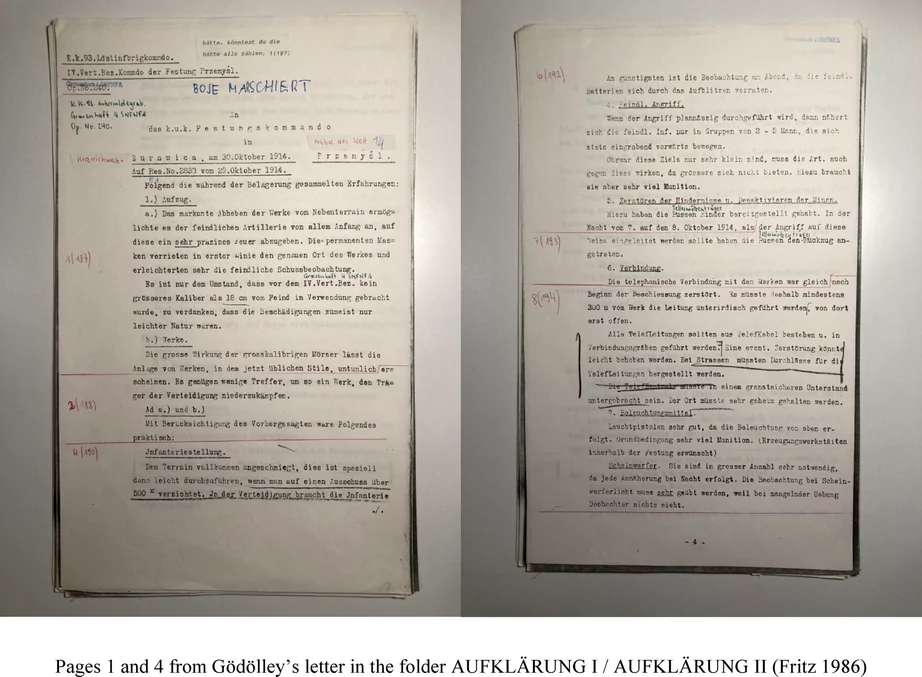

A folder from the Marianne Fritz Archive reveals the status report as an original letter from the Austrian State Archives. It was sent by Captain Gödölley from Przemysl to Colonel Meyer in Zurawica, a municipality to the north, at the end of October 1914. Similar to Vienna’s conversion to Donaublau, Fritz’s handwriting next to the addresses of Zurawica and Przemysl inserts the names “Kranichweib” and “Nabel der Welt” on the first page of the copy. Moreover, in *Naturgemäß*, the status report discards its jargon when it nicknames the enemy. Right by the heading of the letter’s fifth section, which translates to “Destruction of Obstacles a. Deactivation of the Mines”, it reports that the “Tellermützenträger” [plate cap wearers] used cattle to “deactivate” the mines (Fritz 1996, p. 192). The fourth page of the record reveals this compound as a replacement for the designation of the enemy as “Russen” [Russians]. The act of nicknaming as “petrified laughter”, “in which the original act of name-giving still persists” (Horkheimer/Adorno 2002, p. 60), is performed by means of the creation of extended compound words, which is a characteristic of German language. According to Fritz’s poetological approach, it is not the Russians who are given a nickname, but the report is sublated onto another layer, where the enemies are officially named. The renaming in the context of historical uniforms provokes a linguistic reflection on the violence of nominalisation and signifying, thus focusing our intention on the military parlance used by the report.

While Fritz documented every single step of her own working procedures, she did not document the provenance of the records from the Austrian State Archives that she used. This reveals a certain attitude towards the records. Fritz’s copies show how they were not only transcribed but appropriated as a whole for their use in *Naturgemäß*. The title of the chapter, “Boje marschiert” [Boje marches], is written onto the page as well as a motto that is glued onto it. Along the margins, the pagination of the published text can be traced. Each of the two numbers refers to a different narrative unit of *Naturgemäß*. The red lines on the second page mark the insertion of the commander’s speech. The montage constructs an architecture of stories from the archival material, ultimately creating a fortress from language whose complexity is only observable through varying distances.

This brief investigation of basic methods has showed a variety of strategies that provoke a dialogue between different discourses and speaker positions: the montage that makes two genres comment on each other; the idiosyncratic style that not only exposes the characters’ judgements but also positions ourselves within an unresolved situation between the narrator, the character and the reader; the act of nicknaming on different textual layers that highlights both aggressive and emancipative, ‘sublating’ impulses; the appropriation that puts the record as a whole into a fictional context. Fritz shifts words into a new configuration by examining and transferring their former relations, thus forcing an aesthetic reflection upon them. In a letter to her editor, she states that she is concerned with what “documents” do: “exclude”, the “blanks”, the “deleted”, the “unmentioned” or the “surplus” (Fritz 1985 b, p. 7 / Fritz 2010, p. 218).

It becomes apparent that in Fritz’s approach lies an affinity with Jules Michelet’s Romanticism that wants to give a voice to the silenced dead (Rancière 1994, pp. 45 − 6), though with a decisive turn towards archival records and in a different way than Carolyn Steedman outlines in her critique. While arguing that Michelet’s dead “had never existed” (Steedman 2001, p. 152), Steedman states that “historians read for what is not *there*” (Steedman 2001, p. 151). She links the historian’s attitude to a “nostalgia for origins and original referents” that “cannot be performed, because there is actually *nothing there*: […] only silence, the space shaped by what once was; and now is no more” (Steedman 2001, p. 154). But there is a difference between nothingness and a shaped space. The poetic approach allows Fritz to work with shapes without engaging in nostalgia. She examines the shapes of language through the relations they can establish, rather than tracing them back to their origins, to archives or to foundational scenes of different historiographical regimes, as Rancière reconstructs them (Rancière 1994, pp. 25 − 9).

## Conclusion

The case study of Fritz’s novel project *The Fortress* has shown how a complex configuration of different voices and speaker positions are drawn from an appropriation of archival records and shaped by montage, name-giving and forms of narration. The position of the reader is also addressed within this framework. Fritz’s poetic investigation focuses on the modes of address in the archival records, alienating them from their origins and ‘sublating’ them into new contexts. Thus, *The Fortress* is approachable only with the help of archival thinking. While poetic texts have been read in the context of the *archival turn*, the epistemological orientation of those concepts tends to establish a hierarchy among different layers. The poetic structure of *The Fortress*, however, requires us to examine the specific forms in which these layers perform and address one other.

The impulse towards nominal forms and compounds can be observed not only throughout *The Fortress*, but also in the AGFU’s name, *Agency for Indispensability Justifications*, which provides a nickname for an archive. Fritz reads archival records relationally rather than genealogically; the AGFU’s *Aufhebekunst* transfers this poetic form of reading to the *Archive Fritzpunkt*, thus making archiving, rather than the archival records alone, the object of intervention. The AGFU intends to address the archival ethos outside the art world. This ethos, which could be described as a tension between the awareness that “archives are valuable precisely because they have not been created for posterity” (Tschan 2002, p. 191) and the archivists’ necessity to “inject their own values” (Cook 1997, p. 46), is perceived as paradoxical by the AGFU. The poetic act of the AGFU does not intend to create objects of value for future generations, as Barbara Craig assumes when she argues that archivists have to be planners as well as poets (Craig 2001, p. 183). Rather, by ‘sublating’ the addressivity of archival records, *Aufhebekunst* addresses the paradox of planning for a future generation that cannot be adequately anticipated. It creates intricate contexts for records, not to find solutions, but to confront underlying principles. If records “reflect […] specific human activities”, as the Expert Group on Archival Description states as one of the records’ enduring characteristics (EGAD 2023, p. 3), *Aufhebekunst* reflects archiving itself as a specific human activity of the present.

## Data Availability

No datasets were generated or analysed during the current study.

## Footnotes

1 Deleuze 1998, p. 19.

2 Unless otherwise stated in the references, all translations are my own.

3 The “monumental shift” in the digital age, “from a default of forgetting to one of remembering” (Mayer-Schönberger 2009, p. 13), could be read along with one of the AGFU’s catchphrases: what is essential is forgotten in the excess of remembrance. This could also be linked to Mark Fisher’s statement that, in the “nostalgia mode”, we forget our place in time (Fisher 2022, pp. 28 − 9).
